# Characterization of *Scardovia wiggsiae* Biofilm by Original Scanning Electron Microscopy Protocol

**DOI:** 10.3390/microorganisms8060807

**Published:** 2020-05-27

**Authors:** Maurizio Bossù, Laura Selan, Marco Artini, Michela Relucenti, Giuseppe Familiari, Rosanna Papa, Gianluca Vrenna, Patrizia Spigaglia, Fabrizio Barbanti, Alessandro Salucci, Gianni Di Giorgio, Julietta V. Rau, Antonella Polimeni

**Affiliations:** 1 Department of Oral and Maxillo-Facial Sciences, Sapienza University of Rome, Viale Regina Elena 287a, 00161 Rome, Italy; maurizio.bossu@uniroma1.it (M.B.); alessandro.salucci@uniroma1.it (A.S.); gianni.digiorgio@uniroma1.it (G.D.G.); antonella.polimeni@uniroma1.it (A.P.); 2 Department of Public Health and Infectious Diseases, Sapienza University of Rome, P.le Aldo Moro 5, 00185 Rome, Italy; laura.selan@uniroma1.it (L.S.); marco.artini@uniroma1.it (M.A.); rosanna.papa@uniroma1.it (R.P.); gianluca.vrenna@uniroma1.it (G.V.); 3 Department of Anatomy, Histology, Forensic Medicine and Orthopaedics, Sapienza University of Rome, via Alfonso Borelli 50, 00161 Rome, Italy; giuseppe.familiari@uniroma1.it; 4 Department of Infectious Diseases, Istituto Superiore di Sanità, Viale Regina Elena 299, 00161 Rome, Italy; patrizia.spigaglia@iss.it (P.S.); fabrizio.barbanti@iss.it (F.B.); 5 Istituto di Struttura della Materia, Consiglio Nazionale delle Ricerche (ISM-CNR), Via del Fosso del Cavaliere 100, 00133 Roma, Italy; giulietta.rau@ism.cnr.it

**Keywords:** *Scardovia wiggsiae*, *Streptococcus mutans* scanning electron microscopy, biofilm, microbiota, early childhood caries, pediatric dentistry

## Abstract

Early childhood caries (ECC) is a severe manifestation of carious pathology with rapid and disruptive progression. The ECC microbiota includes a wide variety of bacterial species, among which is an anaerobic newly named species, *Scardovia wiggsiae,* a previously unidentified *Bifidobacterium*. Our aim was to provide the first ultrastructural characterization of *S. wiggsiae* and its biofilm by scanning electron microscopy (SEM) using a protocol that faithfully preserved the biofilm architecture and allowed an investigation at very high magnifications (order of nanometers) and with the appropriate resolution. To accomplish this task, we analyzed *Streptococcus mutans*’ biofilm by conventional SEM and VP-SEM protocols, in addition, we developed an original procedure, named OsO_4_-RR-TA-IL, which avoids dehydration, drying and sputter coating. This innovative protocol allowed high-resolution and high-magnification imaging (from 10000× to 35000×) in high-vacuum and high-voltage conditions. After comparing three methods, we chose OsO_4_-RR-TA-IL to investigate *S. wiggsiae*. It appeared as a fusiform elongated bacterium, without surface specialization, arranged in clusters and submerged in a rich biofilm matrix, which showed a well-developed micro-canalicular system. Our results provide the basis for the development of innovative strategies to quantify the effects of different treatments, in order to establish the best option to counteract ECC in pediatric patients.

## 1. Introduction

Early childhood caries (ECC) or baby bottle syndrome [1] is a particularly severe manifestation of carious pathology with rapid and disruptive progression, that affects children between birth and 71 months of age, as defined by the American Dental Association. Epidemiologically, ECC is five times more prevalent than asthma [2], and it is the most frequently experienced chronic disease of global relevance due to its high prevalence, high costs required for treatment and disruption of children′s quality of life [3].

The ECC main cause is the prolonged use of sugary substances (taken mainly at night, when the salivary flow is considerably reduced) combined with the presence of an oral complex microbiota. This latter is rich in acidogenic bacterial species [4], and they metabolize sugars producing lactic acid, which cause dental hard tissues demineralization. In children with ECC, *Streptococcus mutans* (*S. mutans*) frequently exceeds 30% of the microbial flora in the cultivable biofilm plaque [5,6,7]. Other species, identified with a lower percentage, belong to *Veillonella, Granulicatella, Fusobacterium, Lactobacilli, Neisseria, Campylobacter, Gemella, Abiotrophia, Selenomonas* and *Capnocytophaga* [8,9]. Moreover, *Actinomyces* and *Bifidobacterium* were found associated with initial and deep caries, respectively [10,11]. In 2011, a study carried out using anaerobic culture conditions allowed the identification of a newly named species, *Scardovia wiggsiae* [12], which was significantly associated with severe ECC. *S. wiggsiae* was detected also in children without the contemporary presence of *S. mutans*, indicating it*s* exclusive role in this disruptive process [13]. *S. wiggsiae* was classified as an unidentified *Bifidobacterium* species, present within deep dentine caries and in high proportions within infected pulp tissue in children [14]. The most recent literature has shown a significant relationship between the presence of *S. wiggsiae* in the early stages of caries and in pediatric subjects undergoing orthodontic therapy [15,16,17,18]. *S. wiggsiae* has clinical importance in S-ECCs, being a potential risk indicator for the oral health of pediatric patients. Caries formation is closely related to the ability of bacteria to form a biofilm [19]. In the literature, present are only a few preliminary studies on *S. wiggsiae*, but no information from a microbiological and morphological point of view. For this reason, our aim is to investigate the ability of *S. wiggsiae* to form a biofilm, and to provide, for the first time, an ultrastructural and morphological characterization by scanning electron microscopy.

To accomplish this task, we have an additional challenge: since this bacterium and its biofilm have never been observed, it was necessary to implement a suitable protocol that faithfully preserves its ultrastructural architecture and allows an investigation at very high magnifications (order of nanometers) and with the appropriate resolution. Biofilms are extremely hydrated samples, their extracellular polymeric substance (EPS) water content is up to 97% [20]. Thus, biofilms have to be carefully treated for scanning electron microscopy, in order to avoid sample loss, sample shrinkage and collapsing, and other artifacts that could alter the actual morphology [21,22,23,24,25,26,27]. Conventional SEM (high-vacuum and high-voltage electron beam operating conditions) is widely used to provide a detailed biofilm morphological investigation at a high magnification (>10000×). However, conventional SEM protocols, that involve dehydration steps and sputter coating with a metal like platinum, are not so suitable for specimens with such a high content of water as biofilm are. In fact, dehydration and drying processes induce sample loss and artifacts formation, making EPS appear as a fiber network rather than a firm gelatinous matrix [24,28]. Biofilms can be observed by E-SEM without any treatment, in their fully hydrated state, but low-voltage, low-magnification and low-resolution images (low signal to noise ratio) are obtained, and they do not allow their use for a high-resolution ultrastructural characterization. Another available option to observe biofilms is variable pressure scanning electron microscopy (VP-SEM). The VP-SEM chamber condition can be managed at diverse pressure and vacuum settings at an increased humidity, allowing the observation of hydrated samples without coating [23]. In VP-SEM, charging phenomena of sample surface are prevented due to the action of the gas in the specimen chamber: it is ionized by secondary and backscattered electrons and carries away the beam charge formed on the sample surface. Unfortunately, gas presence causes some downsides, the most prominent is the decrease in image resolution, caused by the scattering effect of the gas molecules on the electron beam, and low-resolution images are not suitable to provide a detailed ultrastructural characterization. To obtain a satisfactory ultrastructural investigation of *S. mutans*’ and *S wiggsiae*’s biofilms (the two main bacterial species involved in ECC), different SEM techniques have been compared in our study. To this aim, a new SEM protocol, allowing the retention of the fully hydrated state of the biofilm specimens, has been implemented and a fine ultrastructural characterization of *S. wiggsiae*’s biofilm, never described so far, was carried on.

## 2. Materials and Methods

### 2.1. Bacteria and Culture Conditions

The lyophilized culture of the *S. wiggsiae* DSM 22547 strain (Leibniz Institute, DSMZ-German Collection of Microorganisms and Cell Cultures GmbH) was rehydrated with 0.5 mL of Brain Heart Infusion (Oxoid Ltd., Basingstoke, UK) broth, supplemented with 5 g/L yeast extract, 5 mg/L hemin and 0.5 mg/L vitamin K, (BHIs). The suspension was inoculated on BHIs agar plates and incubated in an anaerobic chamber (90% N_2_, 5% CO_2_ and 5% H_2_) at 35 °C for 48 h. A few colonies of the *S. wiggsiae* DSM 22547 strain were then inoculated in 10 mL of pre-reduced BHIs broth and incubated for 48 h in an anaerobic atmosphere.

A freezer stock of *S. mutans* CCUG 35176, obtained from the Culture Collection University of Göteborg (CCUG), was inoculated on a BHI agar plate and incubated at 37 °C under aerobic conditions. A few colonies of the *S. mutans* CCUG 35176 strain were then inoculated in a tube with 10 mL of BHI broth under vigorous agitation (180 rpm) at 37 °C for 18 h.

The optical density of the *S. wiggsiae* DSM 22547 and *S. mutans* CCUG 35176 cultures were measured with a spectrophotometer Ultrospec 2000 (Pharmacia Biotech Inc, Piscataway, NJ, USA) at 600 nm (OD600) and culture turbidity was adjusted with BHIs broth to OD600= 0.1. Ten milliliters of *S. wiggsiae* DSM 22547 culture was transferred in tubes containing bioactive glass discs and incubated for 120 h in an anaerobic chamber at 35 °C to assess biofilm production. Ten milliliters of *S. mutans* CCUG 35176 culture were transferred in tubes containing bioactive glass discs and incubated for 20 h in an aerobic chamber at 37 °C to assess biofilm production.

### 2.2. Static Biofilm Assay of S. mutans and S. wiggsiae

Biofilm production was quantified using the microtiter plate biofilm assay (MTP) as previously reported [29]. Briefly, the bacterial cultures were diluted to OD600= 0.1 and 100 µL were aliquoted in each well of a sterile 96-well flat-bottomed polystyrene plate. The plate was incubated in an anaerobic atmosphere for 120 h at 35 °C for *S. wiggsiae*, and in an aerobic condition for 20 h at 37 °C for *S. mutans*. After incubation, planktonic cells were gently removed; each well was washed three times with double-distilled water and patted dry with a piece of paper towel in an inverted position. To quantify the biofilm formation, each well was stained with 0.1% crystal violet and incubated for 15 min at room temperature, then each well was rinsed twice with double-distilled water and thoroughly dried. The dye bound to adherent cells was solubilized with 20% (*v*/*v*) glacial acetic acid and 80% (*v*/*v*) ethanol. After 30 min of incubation at room temperature, OD590 was measured to quantify the total biomass of biofilm formed in each well. Each data point is composed of 4 independent experiments, each performed at least in 6 replicates.

### 2.3. SEM Protocols

**Figure 1:**
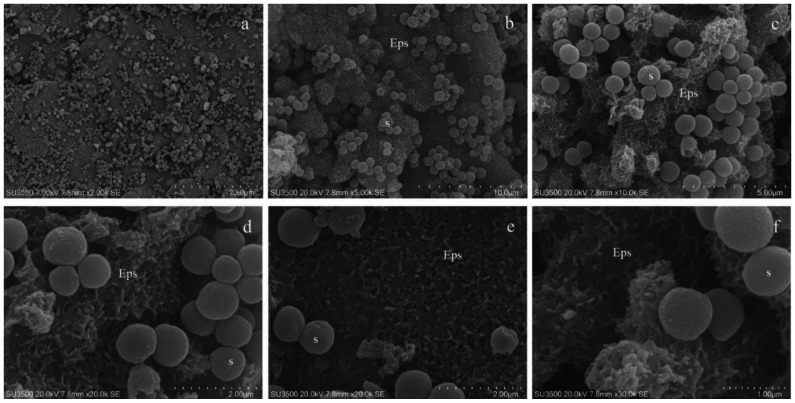
*S. mutans* prepared by conventional SEM procedure. (**a**) SEM, 2000×. At this magnification *S. mutans*’ biofilm appeared as a compact layer and spherical bacterial cells were scattered on its surface. (**b**) SEM, 5000×. At increased magnification biofilm surface show spherical bacterial cells arranged in small groups and Eps reveals its rough aspect. Eps: extracellular polymeric substance, S: *S. mutans*. (**c**) SEM, 10000×. Eps’s forms a canalicular system of compact trabeculae with a spiny surface. Bacterial cells are adherent to the Eps’s surface. Eps: extracellular polymeric substance, S: *S. mutans*. (**d**) SEM, 20000×. Bacterial cells appear irregular, warped and the Eps’s micro-canalicular system is not developed, only superficial holes are visible. Bacterial cells lay down on the Eps’s surface, and they appear naked, without matrix covering. Eps: extracellular polymeric substance, S: *S. mutans*. (**e**) SEM, 20000×. Bacterial cells are sometimes fragmented or indented, and the Eps shows a compact aspect due to the collapse of its fine structure. Bacterial cells, uncovered by the matrix, rest on Eps’s surface. Eps: extracellular polymeric substance, S: *S. mutans*. (**f**) SEM, 30000×. The highest magnification reveals that the Eps’s layers collapse, causing an obstruction of the Eps’s micro-canalicular system. Bacterial cells appear irregular, on the surface, fine granulation due to sputter coating is visible. Eps: extracellular polymeric substance, S: *S. mutans*.

**Figure 2:**
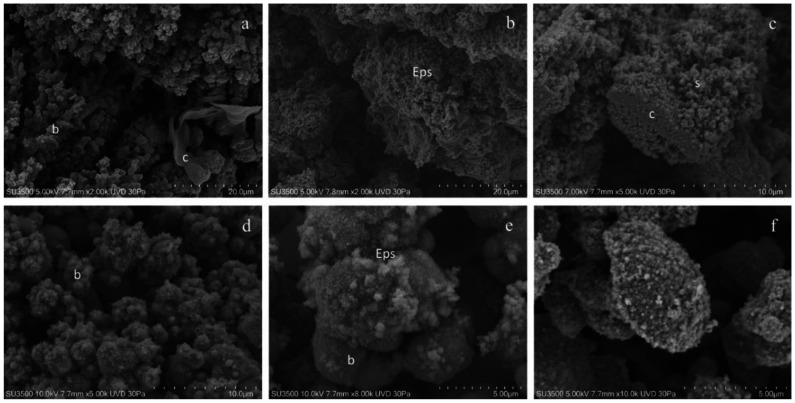
*S. mutans* prepared by conventional VP-SEM procedure. (**a**) VP-SEM 2000X. Biofilm bacterial towers b forming an intricate micro-canalicular system, and sheet of compact matrix c are shown. (**b**) VP-SEM 2000×. Spongy biofilm matrix with the Eps showing no sign of collapse or shrinking; it is well preserved in its hydrated state. (**c**) VP-SEM 5000×. Spongy Eps, s, i**s** developing from a layer of the compact Eps, c. (**d**) VP-SEM 5000×. Bacterial towers b, superficial granulation represents the Eps secretion. (**e)** VP-SEM 8000×. On *S. mutans*’ cells surface, b is clearly visible in the fine graininess of the freshly secreted EPS components. (**f**) VP-SEM 10000×. Bacterial cell secreting EPS, at this high-magnification image, was vague, and focus is difficult to achieve; these are signs of a low signal to noise ratio, the limit of the VP-SEM technique.

**Figure 3:**
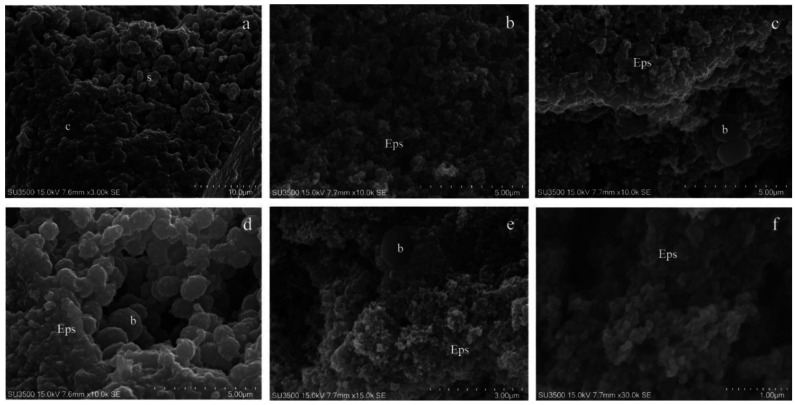
*S. mutans* prepared by the OsO_4_-RR-TA-IL procedure. (**a**) OsO_4_-RR-TA-IL 3000×. The biofilm topography shows both a compact, c, and spongy, s, appearance. Micro-canalicular system is well developed. (**b**) OsO_4_-RR-TA-IL 10000×. At a high magnification, the strength of this technique is revealed, and high-resolution images were obtained without artifacts formation. Eps appears soft, with no signs of collapse or shrinking; micro-canalicular system is intricate. (**c**) OsO_4_-RR-TA-IL10000×. Bacterial cells b appeared surrounded by the Eps, spherical and smooth: no shape alterations were found. (**d**) OsO_4_-RR-TA-IL10000× Bacterial cells **b** are completely embedded in the Eps. (**e**) OsO_4_-RR-TA-IL15000×. High-voltage, high-magnification and high-resolution image of fully hydrated biofilm, where a single bacterial cell b is partially embedded in the Eps. (**f**) OsO_4_-RR-TA-IL 30000×. Very high magnification image confirms the value of this protocol in terms of biofilm three-dimensional structure preservation, until the nanometric level.

Before proceeding with the ultrastructural analysis of *S. wiggsiae*, we tested conventional SEM and VP-SEM protocols on *S. mutans*’ biofilm (Figure 1 and Figure 2), but we were unsatisfied by the results, so we decided to test an original preparation procedure, OsO_4_-RR-TA-IL, (Figure 3), never reported before in the literature, adopting osmium tetroxide (OsO_4_), ruthenium red (RR), tannic acid (TA) impregnation and ionic liquid (IL) drop casting instead of sputter coating. We developed this procedure to combine the advantages of the conventional SEM protocol (image quality, magnification, resolution and long resistance under electron beam) with the advantages of VP-SEM, i.e., reduced preparation time (few steps protocol), minimal sample loss and actual sample structure preservation (achieved avoiding dehydration and drying). To obtain these requirements, the protocol should keep the samples hydrated and not require drying or sputter coating; should allow observation under high-vacuum conditions at acceleration voltages of 1520 kV. High magnifications and high-resolution images obtained under these conditions would allow an ultrastructural characterization without artifacts. To this aim, we decided to use a combination of OsO_4_-RR-TA, reagents already used for a long time in electron microscopy and IL, each one characterized by its own peculiar properties.

RR is a polycationic dye generally used in post-fixation steps, together with osmium or TA. It preserves integrity of negatively charged complex carbohydrates. It is useful to avoid polysaccharide loss in conventional SEM protocols (it is usually high up to 40%) [30]. RR cationic properties enable preservation of EPS polysaccharides and creates electrostatic or ionic links with EPS components, stabilizing the biofilm matrix and avoiding sample shrinking [23,26,30,31,32,33,34,35]. RR for the visualization of extracellular structures with EM was pioneered by Luft [36] and then used on *Staphylococcus aureus* [26,37,38], *Pseudomonas spp*. [39], *Enterococcus faecalis* [34] and *Klebsiella pneumoniae* [34].

Tannic acid reacts with osmium tetroxide and increases lipid retention, forming complexes that link to proteins and carbohydrates [40]. Consequently, they enhance extracellular matrix resistance to mechanical damage during preparation procedures, thanks to a sort of specimen hardening [41,42,43,44]. This method renders the sample itself conductive (not only its surface, as it happens with sputter coating), enhances the image contrast without charging phenomena and allows a three-dimensional observation of its sub-surface structures under higher voltages in comparison with VP-SEM [45].

Ionic liquids are, at room temperature, molten salts with high electronic conductivity and irrelevant vapor pressure [46,47]. These properties allow their use in SEM as a substitute for metal coating [48,49]. We covered biofilm samples with IL, to maintain them wet during the SEM investigation. Even under high-vacuum conditions, ILs resist evaporation, and their use eliminates biofilm dehydration, critical point drying and sputter coating, which contributes to sample preservation.

**Figure 4:**
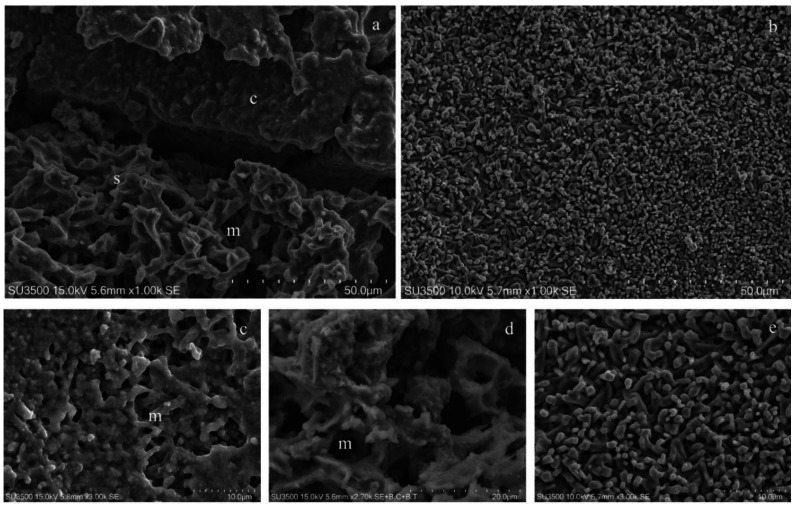
Characterization of *S. wiggsiae*’s biofilm ultrastructural morphology. (**a**) At a low magnification, 1000×, biofilm shows a compact aspect on the top of the picture, c, spongy on the bottom, s, and a microcanalicular system is evident, m. (**b**) Biofilm surface shows a fine granular appearance, 1000×. (**c**) At higher magnifications, a more detailed architecture was distinguishable, compact areas were punctuated with holes, the openings of the microcanalicular system, m. 3000×. (**d**) Spongy areas reveal a trabecular architecture in whose spaces are a canalicular system, m, and unfold, 2700×. (**e**) The granular areas were uneven surfaces due to the presence of digitiform extroversions of the biofilm matrix, 3000×.

**Figure 5:**
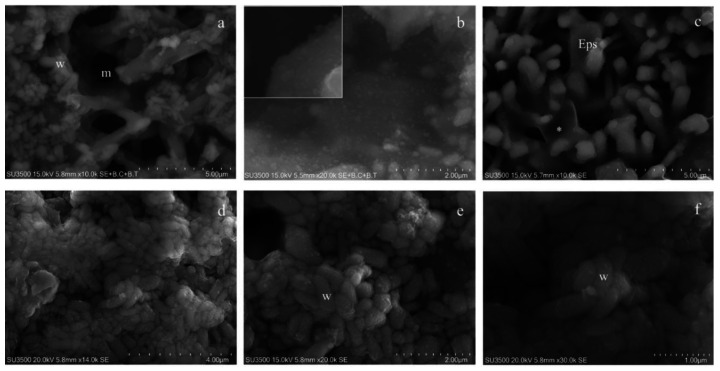
Characterization of *S. wiggsiae* bacterial cells. (**a**) At a very high magnification, 10000×, the biofilm trabecular areas appear populated by clusters of bacteria, w, *S. wiggsiae*, m, microcanalicular system. (**b**) Fine granular aggregates with a cloudy appearance are scattered onto the matrix surface 10000X (inset 35000×). (**c**) Thin matrix sheets, asterisk, connect digitiform extroversions in granular areas creating walls of a canalicular system, 10000×. (**d**) At a very high magnification, bacteria appear as compact clusters of elongated cells without pili or flagella, 14000×. (**e**) A thin veil of hydrated matrix covers bacteria, w, giving them a cloudy appearance, 20000×. (**f**) Very high magnification, 30000×, allows the identification of *S. wiggsiae* bacterial cells, w, as elongated bacteria without surface extroversions.

OsO_4_-RR-TA-IL was evaluated as the most suitable protocol on *S. mutans* (Figure 3, Table 1 and Table 2), so we used the same to characterize *S. wiggsiae* and its biofilm’s ultrastructural architecture (Figure 4 and Figure 5).

Samples of *S. mutans* grown on aluminium disks were processed as reported in Table 1. Samples of *S. wiggsiae*’s biofilm grown for 120 h on bioactive glass discs were processed (after evaluation of results on *S. mutans*) with the OsO_4_-RR-TA-IL protocol. In order to provide accurate measurements of bacterial cell dimensions, we randomly selected several images from 20000× to 35000×. To determine bacterial cell length, we measured, in each selected image, only bacterial cells longitudinally arranged in which both extremities were fully visible (for an overall amount of 100 bacterial cells). To measure the bacterial cell diameter, we used the same images, but we considered only cells with one pole perpendicular to the surface. Once again, we measured 100 bacterial cells. Measurements were carried on by the Image J software and by the SEM image analysis software Hitachi Map 3D (Digital Surf, France). Measure values were statistically analyzed by the MedCalc © software.

## 3. Results

### 3.1. S. mutans Prepared by Conventional SEM Procedure

The first protocol we tested was the conventional SEM technique (Table 1), whose basic steps include fixation in glutharaldehyde, post-fixation in OsO_4_, dehydration in an ascending alcohol series, critical point drying and sputter coating. Each single step could be customized according to peculiar characteristics of the biological sample under examination [50,51,52]. Biofilm has a typical structure based on a delicate three-dimensional network; in order to avoid alteration of this network, we modified the drying protocol by replacing critical point drying with drying in an ascending hexamethyldisilazane series, due to the EPS loss effect of the conventional critical point drying procedure [53].

As shown in Figure 1, conventional SEM has several advantages: it combines a high resolution and depth of field with wide a magnification range (up to 30k, Figure 1a–f); in addition, in our experiments, samples resisted under the high-vacuum condition and the electron beam voltage of 20 kV, allowing the observation over a long period of time. Magnification from 10 to 30k shows downsides of conventional SEM: EPS appeared dense and cells’ shapes were slightly irregular, and the biofilm canalicular system was not evident and its layered structure was crushed. The biofilm shrinkage was due to the collapse of EPS caused by the dehydrating steps [54]. Even if critical point drying was replaced by drying in an ascending hexamethyldisilazane series (more conservative procedure), as stated in [55,56,57], a significant EPS loss would occur.

### 3.2. S. mutans Prepared by Conventional VP-SEM Procedure

VP-SEM allows the imaging of hydrated samples [58,59], such as delicate extracellular matrices, fine collagen fibers and fibrils in ligaments, without dehydration, critical point drying and sputter coating [60]. To image biofilm samples, VP-SEM is an available option [61,62]. It avoids the use of the conventional SEM protocol, which damages the biofilm structure [23,24]. High-vacuum and dry conditions of the SEM chamber are not required: samples observation is possible at different operating conditions of vacuum and humidity [23].

Our images clearly show that VP-SEM technique safeguards EPS better than conventional SEM. The biofilm topography was well-preserved, and the bacterial towers and intricate microcanalicular system were clearly shown (Figure 2a–d). The EPS appeared soft; the aspect of some areas was compact while in others, it was spongy; the bacterial cell shape was regular. Higher magnifications (Figure 2e–f) showed, on bacterial cells’ surfaces, fine graininess of the freshly secreted EPS components. VP-SEM image resolution is lower if compared with SEM images, as well as the sample resistance was shorter under vacuum settings (even if at a low vacuum condition of 30 Pa) and electron beam action (even if 5 kV): this implies a short observation time and appearance of cracking phenomena (Figure 2b,d). Good quality images were obtained up to 10k. At higher magnifications, the signal to noise ratio was lower and the overall image quality dropped significantly.

### 3.3. S. mutans Prepared by OsO_4_-RR-TA-IL Procedure

The EPS’s ability to bind to metals was well known [63]. Metal stains were used in conventional TEM and SEM to increase the EPS polymer’s electron density, thus improving the image resolution [26,34,38]. In conventional SEM, extracellular matrices are preserved using fixatives like glutaraldehyde and osmium tetroxide [34,64,65]. Bacterial EPS is more delicate, and fixatives alone are not sufficient to achieve full preservation or good staining [26,34,38]. To improve these technical deficiencies, we decided to use an OsO_4_-RR solution (tab 1) in the post-fixation step [34,38]. RR is electron dense and, being a cation, binds to the polyanionic polysaccharidic constituents of the EPS [20,34], greatly improving in this way the EPS resolution. To avoid the dehydration procedure and the resulting EPS collapse, we used, after washing the post-fixation solution, an impregnation step with tannic acid. This widely adopted method has several advantages. TA increases lipid retention and binds to proteins and carbohydrates forming complexes; TA reacts with osmium tetroxide [40] and the consequences are enhanced contrast, specimens hardening and an enhancement of extracellular matrices’ resistance to mechanical damage during preparation procedures [41,42,43,44]. This method renders the sample itself conductive without charging phenomena (not only the surface as it is with sputter coating), allowing the three-dimensional observations of its sub-surface structures combined with the use of higher voltages with respect to VP-SEM [45].

After TA impregnation and washing, the sample is still wet and hydrated. To maintain this hydrated state but to observe in a high-vacuum and high-voltage condition, we used ionic liquids drop casting, so our complete procedure was OsO_4_-RR-TA-IL. ILs are molten salts; at room temperature, they exist in the liquid state [66], and they behave as electrically conductive materials [67]. ILs may be added to hydrated samples to replace sputter coating and maintain samples as wet during the SEM observation [46] because they resist evaporation under high-vacuum conditions.

Images of *S. mutans* treated with the OsO_4_-RR-TA-IL procedure showed that the biofilm topography was well preserved and displayed compact and spongy areas; its microcanalicular system was developed and intricate and the EPS texture appeared jagged (Figure 3a,b). *S. mutans* cells were smooth and perfectly spherical in shape; in some areas they emerged from the EPS, in others they were partially embedded, and their surface was covered by the EPS thin layer (Figure 3c,e). The sample sub-surface structure (i.e., different EPS density) is revealed by its intrinsic conductivity (Figure 3d–f). OsO_4_-RR-TA-IL allowed a long observation time up to 30k in a high-vacuum condition, and the biofilm topography was perfectly preserved even at the nanometric level, and the EPS appeared as an intricate three-dimensional network, where no sign of deformation was apparent (Figure 3f).

### 3.4. Evaluation of Best Suitable Protocol for Biofilm Imaging on S. mutans Samples

To evaluate which was the best protocol for the ultrastructural characterization of *S. wiggsiae*, we considered the parameters shown in Table 2 and the informative value given by the overall image quality.

In our opinion, the best suitable protocol was OsO_4_-RR-TA-IL. In fact, this procedure is fast (comparable to VP-SEM protocol) and during the preparation steps, there is a modest sample loss, although there are more steps than in the VP-SEM protocol. This is due to specimens hardening induced by the conductive staining protocol, that enhances the sample structure’s resistance to mechanical damage throughout the preparation procedure. Dehydration and drying are not carried out, preserving the high EPS water content and the actual three-dimensional structure. Moreover, this protocol allows to obtain high-quality images at magnifications up to 30K, and to observe the sample even for 2 h in high-vacuum conditions. In summary, the OsO_4_-RR-TA-IL protocol was preferred over the others because it combines the advantages of the conventional SEM protocol in terms of image quality, magnification, resolution and observation time with the advantages of VP-SEM, like reduced preparation time (few steps protocol), minimal sample loss and actual sample structure preservation (this is achieved avoiding dehydration and drying).

### 3.5. Characterization of Biofilm Formation by S. wiggsiae DSM 22547

*S. wiggsiae* DSM 22547 was investigated for its ability to produce biofilm on a polystyrene plate. Biofilm formation was evaluated at 35 °C in pre-reduced BHI in an anaerobic atmosphere after 120 h of incubation, as described in the Material and Methods section. Based on the quantification of the total biomass at 590 nm, *S. wiggsiae* DSM 22547 can be considered a strong biofilm producer (OD value: 3495 ± 0370).

### 3.6. Ultrastructural Morphological Characterization of S. wiggsiae and Its Biofilm

Observations at low magnifications (1000×) showed a well-preserved biofilm; there was no evidence of shrinking/swelling or artifacts such as unnaturally compacted zones randomly alternated with extremely loose ones. Our samples showed areas with different surface textures: compact, spongy and granular (Figure 4a,b).

At magnifications of about 3000×, the three different textures were further studied. The biofilm surface of compact areas had a rough appearance (Figure 4c) and looked like it was punctuated by small round or oval holes, representing the openings of a microcanalicular system. The biofilm surface of spongy areas (Figure 4d) resembled a trabecular structure, whose scaffold is the biofilm matrix that delimits a labyrinthine canalicular system, a three-dimensional network whose meshes have a larger diameter than that observed in the compact areas.

The granular area’s surface (Figure 4e) appeared as a carpet of digitiform formations (resembling a coral reef) of varying thickness and irregular prismatic shape, which sometimes joined in compact formations.

**Figure 6:**
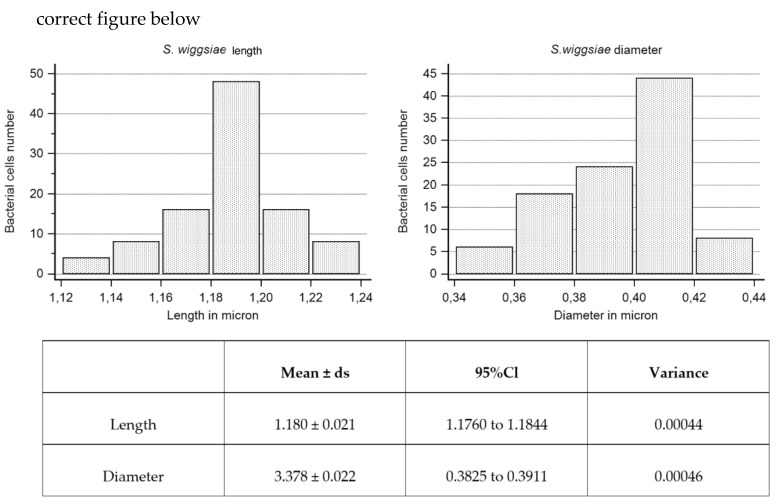
*S. wiggsiae* length and diameter values and their statistical analysis.

Very high magnifications (from 10000× to 35000×) have been used in order to identify bacterial cells (isolated or arranged in clusters) embedded in the matrix bulk. Focusing on matrix trabeculae, in both spongy and granular areas, (Figure 5a,b), it was possible to distinguish their heterogeneous density: in some areas, clusters of bacteria enclosed in the matrix weave were visible; in other areas, the matrix alone, with a different density degree, was present (Figure 5b). The biofilm matrix surface was irregularly dotted with small globular aggregates, visible only at extremely high magnifications (Figure 5b and inset). The digitiform extroversions of variable thickness, which characterize the granular area’s surface, are linked by subtended thin matrix sheets that act as walls for a canalicular system (Figure 5c). In areas with a less dense matrix, zones entirely occupied by bacterial cells were visible. Numerous and intimately packed bacteria were arranged in a carpet-like structure (Figure 5d–f). At a magnification of 14000× (Figure 5d), large clusters of bacterial cells (up to 20µm^2^) were observed. To appreciate the ultrastructural morphology of the individual bacterial cells, and provide accurate measurements, we observed them at very high magnifications, from 20000× to 30000 up to 35000×. Bacterial cells appeared as elongated entities and showed a cigar-like shape, with symmetrically rounded poles. We looked for the presence of surface specializations, like fimbriae, pili or flagella but we did not observe them. The *S. wiggsiae* dimension values are shown in Figure 6.

## 4. Discussion

*S. wiggsiae* and *S. mutans* are the main bacterial species involved in ECC microbiota, an oral affection of early childhood. In the literature, only few and preliminary studies exist on *S. wiggsiae*. Our aim is to provide the first detailed ultrastructural morphological characterization of *S. wiggsiae* and its biofilm. Many microscopy techniques, reported below, are useful in biofilm study. Light microscopy allows the visual identification of the biofilm presence, quantitative assessment of the biofilm biomass and it is useful in combination with transmission electron microscopy or scanning electron microscopy. Confocal laser scanning microscopy enables the visualization and quantification of the biofilm structural parameters, localized cell death and evaluation of antimicrobials’ efficacy on cell viability. Scanning electron microscopy, with its peculiar high-resolution and high-magnification, allows investigating very fine details of the biofilm surface and its spatial structure. It supports in evaluating how antibiofilm drugs modify both the biofilm structure and ultrastructure of bacterial cells. In addition, SEM images, analyzed by dedicated software, can give quantitative information on biofilm formation kinetics. Cryo-SEM provides information on the topography and structure of the bacterial glycocalyx; using freeze-fracture methods, inner structural details of biofilm are visible. Cryo-SEM is a useful technique to investigate liquid or semi-liquid samples. Environmental-SEM allows the imaging of samples in their natural hydrated state; it is suitable for the dynamic study of gas–liquid–solid interactions both in situ and in real time. Focused ion beam-SEM is useful in studies on environmental biofilms, to investigate the sub-surface structure of biofilms, and to provide three-dimensional reconstructions. Lastly, atomic force microscopy permits quantitative in situ imaging and biofilm analysis; it is usually adopted to confirm findings obtained with other techniques. It helps to study adhesion forces between a biofilm and its substratum, as well as the biofilm topography. Each one of these techniques has its own advantages and disadvantages—for review, see [68]. Based on what is reported above, in order to characterize both the *S. wiggsiae* morphology and biofilm structure, we decided to use scanning electron microscopy, which reaches the highest resolution over other techniques, allows resolving fine surface details and reaches high magnifications. To counteract SEM protocol disadvantages (dehydration, drying and coating procedures, high-vacuum and high-voltage operating conditions), we elaborated an original protocol, OsO_4_-RR-TA-IL, never reported in the literature.

In order to evaluate which was the best protocol, we have considered the parameters shown in Table 2. Sample loss during the preparation procedure is a key factor: if the yield of the protocol is low, it means that the procedure mechanically damages the sample. In fact, it can happen that the sample amount is insufficient to allow a morphological investigation at different magnifications and in different areas of the sample, and that the sample is damaged and not suitable to be used. Another important parameter is the sample resistance in the vacuum environment of the specimen chamber and under the electron beam action. Obviously, we have set the recommended operational parameters for each type of observation, based both on our long professional experience [69,70,71,72] and on the recommendations in the literature. Then, we considered the achievable magnifications; to characterize a sample never observed before, it is necessary to reach magnifications in the range 20–30K, in order to show the minutest ultrastructural details. Finally, we have considered the overall quality of the images in terms of definition, intrinsic contrast, absence of charging phenomena and the possibility to observe the sub-surface. These are essential criteria, being an ultrastructural morphological characterization based on the evaluation of high-quality images without artifacts (induced by both the preparation procedures and the observation conditions set in the electron microscope). Considering the above-mentioned, in order to investigate *S. wiggsiae* and its biofilm, we have chosen the OsO_4_-RR-TA-IL protocol, a fast-experimental procedure, comparable with the VP-SEM protocol, which does not involve excessive sample loss. This protocol assures the absence of the dehydration and drying steps, which modify the actual biofilm’s three-dimensional structure. The intrinsic conductivity of the sample, due to the use of RR and TA and implemented by ILs, gives the opportunity to observe for a long time under high-vacuum conditions, using 15–20kV voltages; this allows high quality imaging at high magnifications, needed for an ultrastructural biofilm characterization. The high-magnification and high-resolution images of the wet samples in high-vacuum and high-voltage environmental conditions, showed in this paper, provide the first ultrastructural description of the *S. wiggsiae* biofilm and of single bacterial cells. The biofilm aspect at SEM is strictly related to accelerating voltages; only using higher accelerating voltages is it possible to visualize the sub-surface structure of the biofilm because electrons can penetrate deeply into the specimen. Using our conductive staining procedure and ILs, both surface and sub-surface structures were imaged. We were able to show that *S. wiggsiae* produces a compact or spongy biofilm, whose trabecular structure delimits a labyrinthine three-dimensional canalicular system. In areas where the biofilm was less dense, numerous and intimately packed elongated bacteria were visibly embedded in the matrix. Cells appeared smooth, elongated and symmetrically rounded at the poles, without surface specializations like fimbriae, pili or flagella.

Our morphological investigation provides the basis for future studies on the treatment and/or prevention of ECC. In fact, the characterization of the normal *S. wiggsiae* bacterial cells and biofilm morphology will allow to evaluate the effects of different treatments, in order to establish the best therapeutically suitable options for pediatric patients.

## 5. Conclusions

This paper reports the first ultrastructural characterization of the *S. wiggsiae* biofilm by an original scanning electron microscopy protocol, the OsO4-RR-TA-IL treatment. The biofilm appeared as an intricate three-dimensional architecture of Eps trabeculae, in which a complex micro-canalicular system was developed. *S. wiggsiae* appeared as an elongated bacterium, without pili or fimbriae. It forms a bacterial cells cluster embedded in the Eps scaffold. The findings of our present study will be useful to test in vitro the way of action of new strategies to prevent and/or treat ECC in pediatric patients.

## Figures and Tables

**Table 1:** Summary of different treatments steps and operating conditions of observation.

		Protocols		
	Steps	**Conventional SEM**	**VP-SEM**	**OsO_4_-RR-TA-IL**
1	Fixation	Glutharaldehyde 2,5% in PB 0.1 M pH 7.4 at least 48 h		
2	Washing	10 min ×2 times in PB 0.1 M pH 7.4		
3	Postfixation	OsO_4_ 2% 1 h	OsO_4_ 2% in 1 h	OsO_4_ 2% + RR 0.2% 1:1 solution, 1 h
4	Washing	10 min ×2 times in dH_2_0 10 min ×2 times in dH_2_0 10 min ×2 times in dH_2_0		
5	Impregnation	None	None	Tannic Acid 1% in d H_2_0 30 min
6	Washing	continues from 2	continues **from** 2	10 min ×2 times in dH_2_0
7	Dehydration	Ascending ethanol series	None	None
8	Drying	Ascending HMDS ^1^ series	None	None
9	Pt Sputter coating	15 mA, 2 min	None	Replaced by IL
	Operating conditions	1520– kV, high vacuum	510–kV 30 Pa	1520– kV, high vacuum

^1^ HMDS: Hexamethyldisilazane, HN[Si(CH3)3]2T.

**Table 2:** Parameters considered in the protocol selection.

Parameters	Protocols		
	Conventional SEM	VP-SEM	OsO_4_-RR-TA-IL
Procedure time	2 days	1 h and 30 min	2 h and 10 min
Sample loss	Steps produce sample loss of about 60%	about 20%	about 20%
Dehydration and drying	yes	None	None
Pt sputter coating	yes	None	Replaced by IL
Resistance in vacuum	Excellent, it is possible to observe for hours	Good for 1 hour	Excellent, it is possible to observe for hours
Operating conditions	1520 kV, high vacuum	510 kV 30 Pa	1520 kV, high vacuum
Image magnification	Good up to 40k	Good up to 10k	Good up to 30k
Image quality	Excellent up to 30k	Good up to 8k	Excellent up to 30k
